# Ordered Mesopore Channels of SBA-15 for Contaminant Adsorption: Characterization, Kinetic, Equilibrium, and Thermodynamic Studies

**DOI:** 10.3390/molecules30051040

**Published:** 2025-02-24

**Authors:** Francisco Emanuel da Silva, Marcela Pires Spaolonzi, Melissa G. A. Vieira, Sibele B. C. Pergher

**Affiliations:** 1 Institute of Chemistry, Universidade Federal do Rio Grande do Norte, Senador Salgado Filho Av., 3000, Natal 59078-970, RN, Brazil; fran.hemanuel22@gmail.com; 2 School of Chemical Engineering, Universidade Estadual de Campinas, Albert Einstein Av., 500, Campinas 13083-872, SP, Brazil; marcela_spaolonzi@hotmail.com (M.P.S.); melissagav@feq.unicamp.br (M.G.A.V.)

**Keywords:** SBA-15, adsorption, emerging contaminants, ciprofloxacin, bisphenol A, losartan

## Abstract

SBA-15 is used in various processes, including adsorption, due to its textural properties, mesoporous channels, and silanol groups on the surface. These characteristics make it a promising material for the adsorption of emerging contaminants. This work evaluated the potential use of SBA-15 for the adsorption of bisphenol A (BPA), ciprofloxacin (CIP), and losartan (LS). This study showed that the material has highly ordered mesoporous channels and silanol groups on the surface, which influenced the affinity tests. SBA-15 exhibited the highest adsorption capacity (0.1317 mmol g^−1^) and removal percentage (60%) for CIP among the contaminants assessed. The adsorption mechanism was elucidated, revealing different interactions for each molecule. The kinetic curves for CIP adsorption indicated that the process reached saturation in 20 min, the equilibrium isotherm showed the highest adsorption at 15 °C, and the thermodynamic study shows an exothermic behavior and spontaneous process. The simplified batch design estimated that 27 g of SBA-15 is required to treat 10 L of 0.2 mmol L^−1^ initial CIP concentration solution and achieve 90% adsorption removal. This material demonstrated satisfactory performance in absorbing emerging contaminants.

## 1. Introduction

The constant increase in the world’s population has brought major problems of water pollution with emerging contaminants. These contaminants can be produced by plastics, surfactants, or pharmaceutical industries and are not efficiently removed from water treatment plants [1]. Several methods have been developed for the treatment of emerging contaminants, encompassing physical, chemical, and biological approaches [2].

Physical treatments, such as filtration, sedimentation, and membrane separation processes, are widely used due to their simplicity and speed [3]. However, these methods have significant limitations in effectively removing molecules at microscopic levels, particularly at the very low concentrations commonly found in emerging contaminants [4,5]. Furthermore, these processes often only transfer the contaminant to another phase without effectively degrading it, which can lead to the generation of secondary waste and additional environmental impacts [6,7].

Chemical and biological methods also have significant disadvantages. The introduction of chemicals in the treatment can generate unwanted by-products and, in some cases, worsen the contamination, in addition to altering essential parameters, such as the pH, making the water reuse process difficult [8,9]. Biological processes, however, although promising, may have limitations, such as difficulties in treating complex mixtures of contaminants or even generating secondary problems, such as excessive biomass growth or the release of metabolites that can be equally harmful [10,11].

The adsorption process using solids appears to be one of the most promising alternatives. This technique allows efficient separation between phases, offers excellent results in contaminant removal, and is easier to operate, with the potential for large-scale application in a sustainable and economical manner [12,13].

The SBA-15 emerges as an alternative adsorbent for this process. It is a material with a 2D hexagonal structure, a pore diameter between 5 and 30 nm, and is microporous, which is important for thermal stability [14,15]. This material has a larger surface area, pore diameter, and chemical and thermal stability in processes with a pH close to 4 and 8. This is due to the silica structure with silanol groups for surface interactions [16]. However, little is known about this material’s efficiency in absorbing emerging contaminants without surface functionalization and about how BPA, CIP, and LS molecules interact with the silica surface and any functional groups present.

This study aimed to assess how the SBA-15 material can effectively be used as an adsorbent in adsorption processes. For this purpose, an affinity test was performed to select the best adsorbate–adsorbent system. In addition, adsorption kinetics and equilibrium studies were performed, and the possible adsorption mechanisms were elucidated.

## 2. Results and Discussion

### 2.1. Characterization of the Nanostructured Mesoporous Material SBA-15

**Figure 1:**
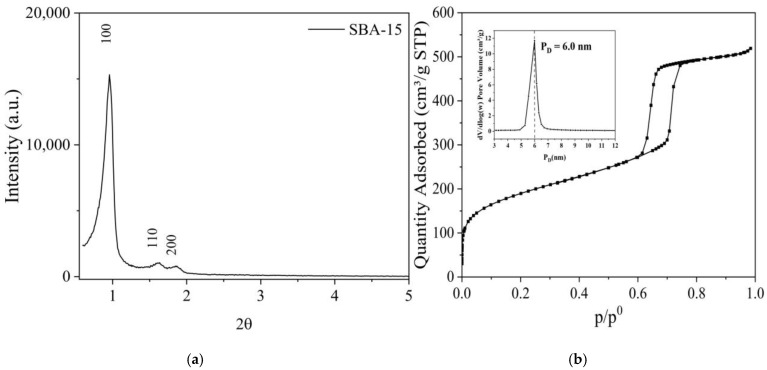
X-ray diffraction (**a**) and N_2_ physisorption for SBA-15 (**b**).

Figure 1a shows XRD for SBA-15 at a low angle, ranging from 0.5° to 5°, where three peaks are observed. The first peak of higher intensity corresponds to the (1 0 0) plane, while the second and third correspond to the (1 1 0) and (2 0 0) planes, respectively [17]. These peaks are characteristic of 2D hexagonal mesoporous structures, as shown in Figure 1b, which exhibits a type IV isotherm with H1-type hysteresis [18]. Table 1 presents the textural properties, with a surface area (S_BET_) of 688.7 m^2^ g^−1^ and a mesoporous volume of 0.731 cm^3^ g^−1^. However, the material also contains micropores, with a volume of 0.073 cm^3^ g^−1^. Therefore, the external area (representing the mesoporous area) is 504.8 m^2^ g^−1^, and the pore diameter is 6.0 nm.

**Figure 2:**
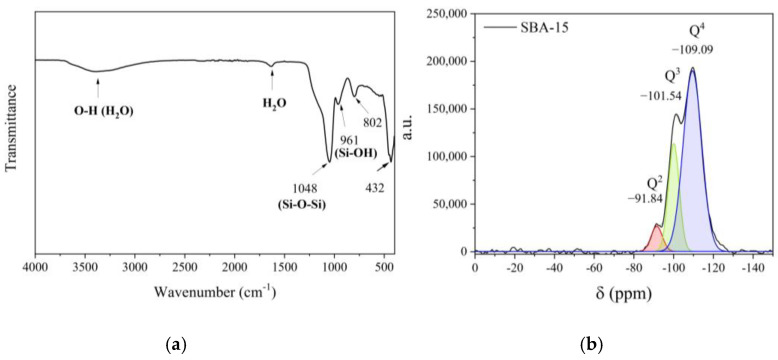
FTIR (**a**) and NMR (**b**) for SBA-15.

Figure 2a (FTIR spectrum) shows a band at 432 cm^−1^ representing the Si-O-Si bonds to condensed structure, and the peaks at 802 and 1048 cm^−1^ are symmetric and asymmetric stretching vibrations, respectively. The peak at 961 cm^−1^ is attributed to the silanol group (Si-OH) present in the material structure. Figure 2b shows the ^29^Si NMR with Q^4^ (−109.09 ppm) peak related to the siloxane group (Si-O-Si). The peaks of Q^3^ (−101.54 ppm) and Q^2^ (−91.84 ppm) are from silanol group (Si-(OH)_2_) and isolated silonals (Si-OH), respectively [19]. Table 2 presents the signal parameters for NMR. Q4 presents the largest area value (63.79%). The areas of Q^3^ and Q^2^ present area values of 30.48% and 5.73%, respectively. The silanol group has higher concentrations than isolated silonals, and the surface has more silanol bridges than isolated ones.

**Figure 3:**
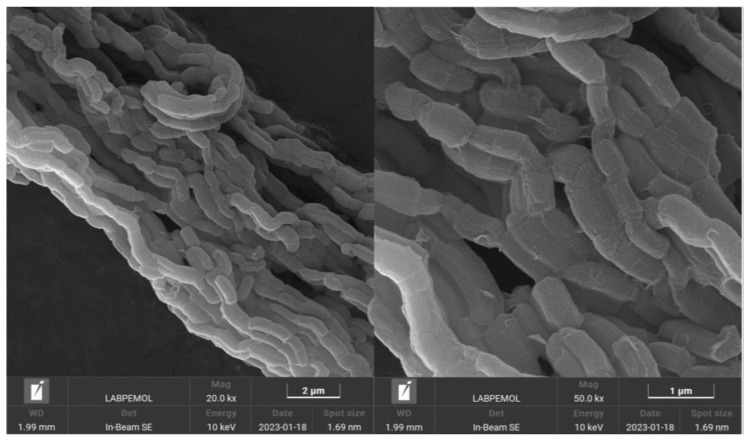
SEM micrographs for SBA-15 material.

Figure 3 shows the SEM images for the SBA-15 material. The fiber morphology consists of smaller particles. This is important for continuous mesoporous channels in all structures. The entire material is organized in one direction with a rough surface. Previous works have shown the importance of morphology for intrachannel diffusion, such as in platelets that have short and striking textural properties [20]. Fiber morphology provides high performance in adsorption processes thanks to its accessibility when compared to other morphologies, such as spheres that have curved mesoporous channels and obstructions in their pores [21,22]. The EDS spectrum (Figures S1 and S2 in the Supplementary Materials) was analyzed to determine the chemical composition of the material. The results confirmed that the material is composed exclusively of Si and O species, indicating its constitution as SiO₂, consistent with the findings reported in the literature [23].

### 2.2. Affinity and Adsorption Mechanism

**Figure 4:**
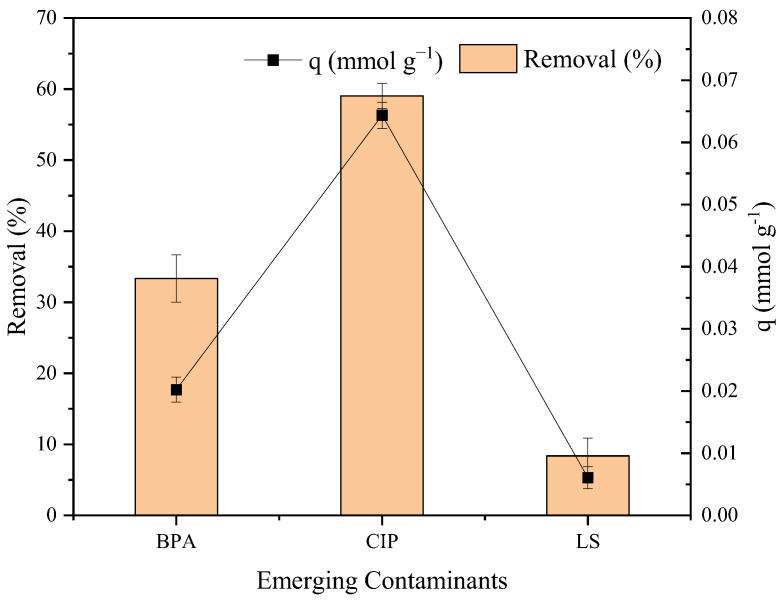
Affinity test for BPA, CIP, and LS on SBA-15.

Figure 4 shows the affinity test for the adsorption of bisphenol A (BPA), ciprofloxacin (CIP), and losartan (LS) on SBA-15. CIP presents the best performance, with a maximum %R of 60% and an adsorption capacity (*q*) of 0.0644 mmol g^−1^. BPA presents a removal of 33% and *q* of 0.0202 mmol g^−1^. LS presents lower values than the other two molecules, with a removal percentage of 8% and a *q* of 0.0060 mmol g^−1^.

Compared to other materials, SBA-15 exhibits satisfactory adsorption performance. For example, clinoptilolite zeolite and pumice achieved removal rates of 51% and 25%, respectively, for CIP [20]. Activated carbon, previously tested for this contaminant, showed a value to *q* of 0.0111 mmol g^−1^ [21]. Kaolinite material shows 0.02 mmol g^−1^ for ciprofloxacin [22]. This may indicate that the material used in this work performed excellently for this contaminant.

**Figure 5:**
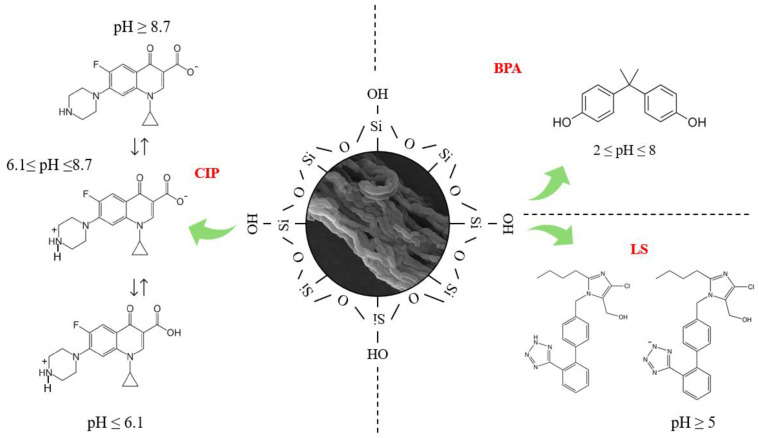
Adsorption mechanism of BPA, CIP, and LS on SBA-15.

Figure 5 shows the adsorption mechanism for BPA, CIP, and LS in the mesoporous channels of SBA-15. All experiments were conducted in ultrapure water at a pH of 5 without any pH control. It is important to understand how each molecule interacts with SBA-15. BPA remains unchanged at pH levels between 2 and 7, and hydrogen bonds are formed through interaction with silanol groups (Si-OH) present on the adsorbate surface [24]. LS carries a negative charge at a pH close to 5, interacting with the siloxane group due to its partially positive charge on Si. However, repulsive forces arise between LS and the silanol group due to the negative charge on OH, which hinders the adsorption process [25]. CIP can exhibit three distinct forms according to the solution’s pH. At a pH ≤ 6.1, it acquires a negative charge due to the amine group being in protonated form (cationic form) [26]. At a pH ≥ 8.7, it is in an anionic form due to the deprotonation of the carboxylic acid group in the structure of the molecule [27]. Between a pH of 6.1 and 8.7, it is in an isoelectric state because CIP is a zwitterionic compound [28]. At a pH close to 5, this molecule presents electrostatic interactions with Si-O-Si and Si-OH, which have a great impact on the adsorption process, allowing adsorption at different sites.

**Figure 6:**
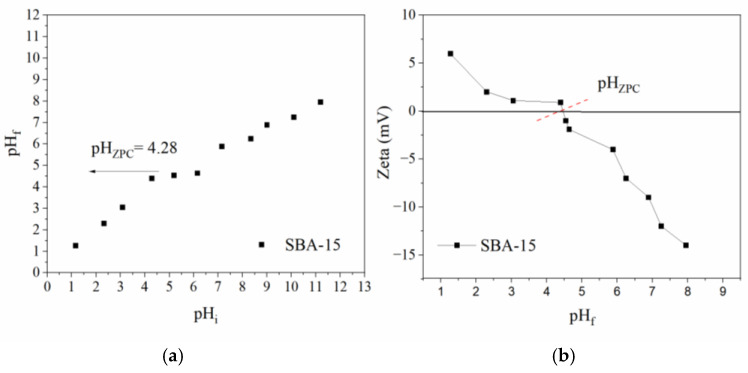
Determination of pHzpc with pHi (pH initial) and pHf (pH final) (**a**); determination of zeta potential (**b**) to material SBA-15.

Figure 6a shows the determination of SBA-15′s zero-point charge using NaOH as an electrolyte to determine ionic strength and pH, with a range of 1 to 11. The plateau is observed at a pH of 4.28, this indicates the pH_ZPC_ region. Some studies have indicated the regions at a pH of 4 [29], and a pH of 5.2 in a material with different synthesis parameters [30]. This material presents a positive charge in the pH region of 1 to 4 and a negative charge in the pH region of 5 to 8 (Figure 6b). Previous works have shown the same regions for this charge on the SAB-15 surface [31,32]. This is important to the adsorption process; the CIP presented higher adsorption in the mesoporous channel at a pH close to 5, which indicates that the molecule has a positive charge and SBA-15 presented a partially negative surface, causing an attraction between the charges.

Therefore, the best adsorbent/adsorbate system was chosen to continue this study, with the evaluation of kinetic and equilibrium tests, in addition to thermodynamic calculations and simplified batch design. The chosen system was the one formed by the antibiotic ciprofloxacin and SBA-15.

### 2.3. Kinetic Study to Adsorption of CIP

**Figure 7:**
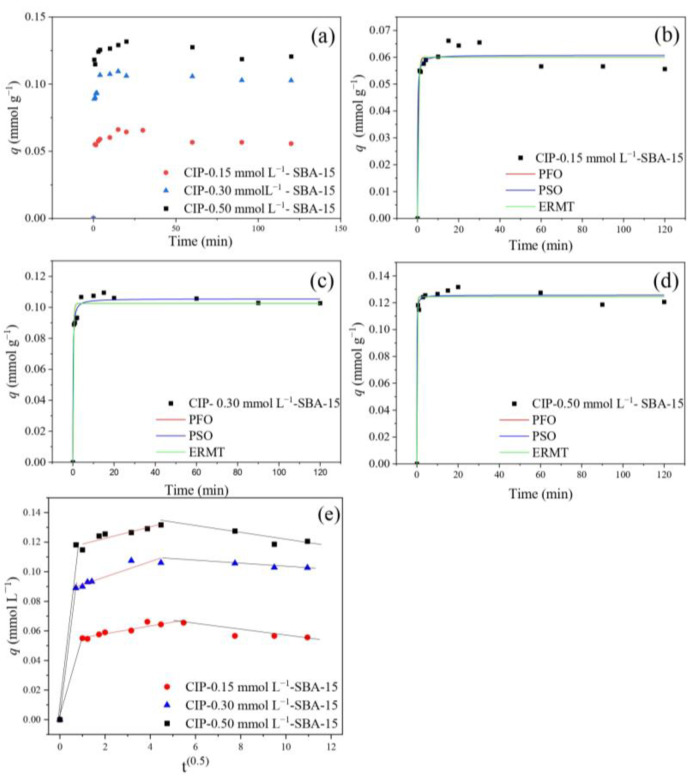
Kinetic curves of CIP adsorption on SBA-15 at 0.15, 0.30, and 0.50 mm L^−1^ (**a**); Models for 0.15 (**b**), 0.30 (**c**), and 0.50 mmol L^−1^ (**d**); IP model (**e**).

Figure 7 illustrates the curves of CIP adsorption on SBA-15 at 0.15, 0.30, and 0.50 mmol. L^−1^. The system exhibited an equilibrium time of 20 min, showing excellent performance at the beginning of adsorption and continuing until the saturation plateau. The adsorption capacity has a linear growth as the initial concentration increases. The q at equilibrium for 0.15, 0.30, and 0.50 mmol L^−1^ is 0.0644, 0.1061, and 0.1317 mmol g^−1^, respectively.

Regarding the time to reach the saturation plateau, the material in this study demonstrated faster results compared to others [33,34,35,36]. Wu et al. [37], for example, studied the use of mixed oxides, which presented times ranging from 60 min to 600 min. Similar results were found by Tran et al. [38] with a chitosan/biochar hydrogel sphere. The authors also found times to reach the equilibrium plateau between 60 and 600 min. Montmorillonite was studied by Wu et al. [39] and presented an equilibrium time of 60 min at different initial concentrations, indicating slower adsorption.

Figure 7b–e show the fit to the PFO, PSO, ERMT, and IP models. Table 3 shows the model parameters applied to experimental data. The PFO model presented an estimated adsorption capacity at equilibrium (*q_e_*) of 0.06, 0.102, and 0.122 mmol g^−1^ for initial concentrations of 0.15, 0.30, and 0.50, respectively, and the PSO model presented 0.061, 0.106, and 0.126 mmol g^−1^ for these concentrations. The PSO model presented the best R^2^_Adj_; however, the PFO model presented lower AICc values (corrected Akaike criteria). This may indicate that adsorption may occur through chemical interactions at the active sites on the SBA-15 surface. Furthermore, physical adsorption is also present in the process, with intraparticle diffusion in the mesoporous channels [40]. The mean relative deviation (MRD) was calculated to obtain statistical data from the presented models.

Some models are important to describe adsorption control mechanisms. For this purpose, the IPD and EMRT models were adjusted to the experimental data. The IPD model presents multilinearity and the second region is the most important since it represents diffusion within the pores. The lowest value for R^2^_Adj_ indicates that the diffusion process cannot describe the limiting phase [41]. The EMRT presents higher values for R^2^_Adj_ for all concentrations, indicating a satisfactory adjustment. The K_TM_ (decay of the diffusion rate of the outer film) increases with increasing initial concentration. This may suggest that there is a resistance in the outer film that limits the adsorption process [37,42]. This behavior aligns with the increasing thickness of the diffusion boundary layer at higher concentrations, which impacts the adsorption rate.

Analysis of the fitted models shows that although chemical interactions at the active site on the material surface are an important factor, the resistance to external mass transfer also affects the adsorption process, especially at higher initial concentrations, where the effect of the diffusion boundary layer becomes evident. The choice between models should therefore consider both the nature of the adsorbent interactions and the effects of the physical transport.

### 2.4. Equilibrium Isotherm

**Figure 8:**
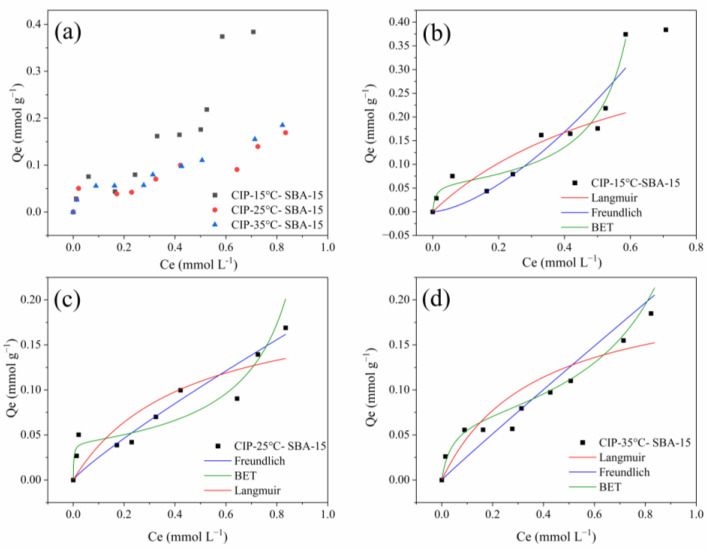
Equilibrium for CIP adsorption on SBA-15 at 15°, 25°, and 30° C (**a**); models fitted to 15° C (**b**); 25° C (**c**); and 35° C (**d**).

Figure 8 presents the graph of the isotherms obtained at 15 °C, 25 °C, and 30 °C. This adsorption process presents an increase according to temperatures, which may indicate that the process exhibits exothermic behavior [43]. Figure 8b–d show the models fitted.

All parameters of models applied to experimental data are described below (Table 4). The isothermal profiles suggest that the material may not be fully saturated and higher q_L_ (Langmuir model) may confirm this when compared to the experimental *(q_max_*) [44]. The *n* (Freundlich parameter) was <1 for 25 and 35 °C and >1 for the temperature of 15 °C. This may indicate that the interactions have lower energy at higher temperatures and physical interactions may be present. However, for the lowest temperature evaluated, there is higher energy, and chemical interactions may be involved [45]. The B.E.T. model shows a satisfactory R^2^Adj for all temperatures (>0.9), suggesting multilayers with diverse interaction sites. The q_b_ value (monolayer) was between 0.0059 and 0.068 mmol g^−1^. However, from the AICc values, it is possible to observe that the B.E.T. model is the one that best describes the system at a temperature of 15 °C.

The Langmuir model clearly does not describe the adsorption process as presented by the equilibrium curves. Table 5 presents a comparison of *q_L_* (Langmuir model) across distinct adsorbents, along with all the adsorption parameters for CIP adsorption. It can be noted that this work presents the shortest equilibrium time, only 20 min, and achieves a promising q_L_. A similar material, MCM-41, was evaluated by Lu et al. [41], and presented a time of 60 min and a q_max_ value of 0.210 mmol g^−1^, close to that achieved by the evaluated SBA-15. Carbon-based materials were also evaluated for CIP removal. In general, these materials took a long time to reach equilibrium and achieved lower *q_L_* values.

**Table 5:** Comparison of Langmuir *q_L_* and adsorption parameters for different adsorbents.

Material	*q_L_* (mmol g^−1^) ^1^	Dosage (g L^−1^)	Equilibrium Time (min)	Initial Concentration (C_0_) (mmol g^−1^) ^1^	Temperature (°C)	Reference
SBA-15	0.207	1.5	20	0.01–1.50	25	This work
MCM-41	0.210	1.5	60	0.03–0.90	25	Lu et al. [41]
Functional silica aerogel	0.176	1.5	90	0.015–0.38	25	Sert Çok et al. [46]
Bulk pomegranate peel	0.016	10	240	0.03–0.36	25	Hamadeen et al. [47]
Activated Carbon (Mangosteen Peel)	0.090	0.75	60	0.15–1.20	25	Tran et al. [38]
Chitosan/biochar hydrogel beads	0.242	5	600	0.015–0.50	30	Afzal et al. [48]
Chitosan/Kaolin/Fe_3_O_4_	0.144	0.75	60	0.03–0.60	25	Ma et al. [49]

^1^ Conversion of mmg g^−1^ to mmol g^−1^.

Table 6 exhibits the thermodynamic parameters computed from experimental isothermal data at 15°, 25°, and 35°. This process exhibits exothermic behavior, characterized by heat dissipation [50]. The enthalpy change (ΔH°) for the adsorption process is negative (−89.208 kJ mol⁻¹), indicating energy release [51,52]. The adsorbed state of ciprofloxacin (CIP) is more ordered due to the loss of molecular degrees of freedom during adsorption, resulting in a negative entropy change (ΔS°) [53]. The process is spontaneous, as indicated by the negative Gibbs free energy (ΔG°) values, which range from −29.675 to −27.819 kJ mol⁻¹, a common characteristic of exothermic processes [54]. Based on these characteristics, the adsorption of CIP on SBA-15 exhibits ΔH° < 0. This behavior suggests that at higher temperatures, there is a reduction in the *q* of the material, a typical phenomenon in exothermic processes. The negative activation energy (E_a_) can be interpreted as a reflection of the increased efficiency of the system at lower temperatures.

### 2.5. Simplified Batch Design for CIP-SBA-15

**Figure 9:**
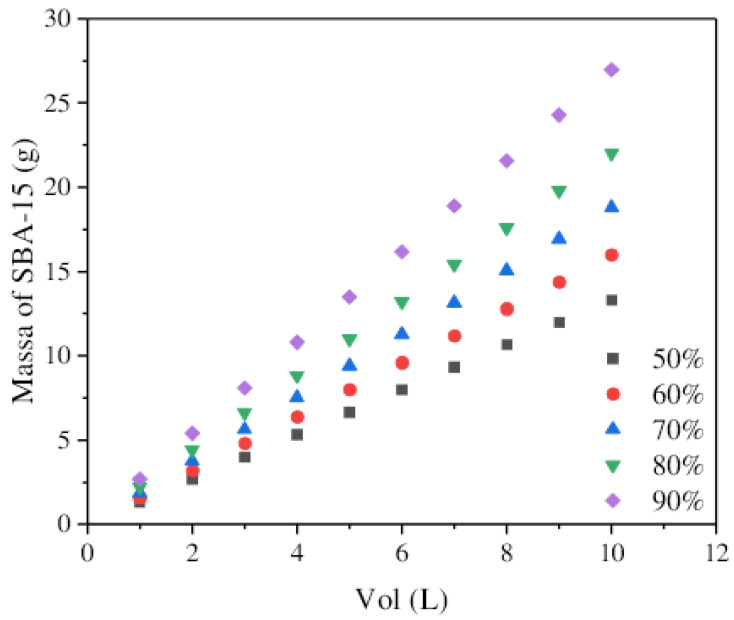
Simplified batch design for CIP system on SBA-15 with different removal percentages.

The simplified batch design was carried out considering the application of solution volumes ranging from 1 to 10 L, with a CIP concentration of 0.2 mmol L^−1^ and an R% efficiency between 50% and 90%. The calculations were based on the B.E.T. model. Figure 9 shows the mass required for the conditions evaluated.

Linearity is observed, and expected, concerning the mass of adsorbent required as the desired removal percentage and the increase in the volume to be treated. The maximum condition, reaching 90% removal and 10 L of treated solution, requires 27 g of SBA-15. In addition, the material used in this work has a lower synthesis price when compared to commercial SBA-15 or even other adsorbent materials, such as MCM-41, which presented adsorption capacity like SBA-15. The synthesis cost of the SBA-15 synthesized is approximately USD 8/g. For commercial SBA-15 (Sigma-Aldrich^®^_,_ St. Louis, MO, USA) it is around USD 38/g, and for MCM-41, it is around USD 15,960/g (Sigma-Aldrich^®^_,_ St. Louis, MO, USA). Compared to these materials, SBA-15 has excellent performance and market value.

## 3. Materials and Methods

### 3.1. Materials

For the synthesis of SBA-15, Pluronic^®^ P123 (Poly(propylene glycol)-block-poly(ethylene glycol)-block-(propylene glycol, Mw~5800), HCL (hydrochloric acid P.A., 37% by weight), ultrapure water and tetraethyl orthosilicate (TEOS, P.A., purity > 98.0%), and NaOH (sodium hydroxide P.A., purity > 99%), all obtained from Sigma-Aldrich (St. Louis, MO, USA), were used. The synthetic solutions for the adsorption tests were prepared with ciprofloxacin (EMS S/A, ≤99%; Hortolândia, SP, Brazil), losartan (Purifarm, ≥99%; São Paulo, SP, Brazil), bisphenol A (Sigma-Aldrich^®^, ≥99%, St. Louis, MO, USA), and ultrapure water.

### 3.2. Synthesis of Mesoporous Silica Nanostructure

The SBA-15 material was synthesized using the methodology of Zhao et al. [55]. The P123 (16.3 g) was dissolved in water (519.2 g) and HCl (96.7 g) for 3 h at 35 °C. The 34.8 g of TEOS was added to the solution and kept stirring for 20 h. Afterwards, the mixture was added to the Teflon autoclave at 100 °C for 24 h, then filtered until reaching a pH of 7 and calcined at 550 °C for 6 h in 2° min^−1^.

### 3.3. Adsorbate Characterization

X-ray diffraction (XRD) was used to characterize the ordered structure, using a range of 0.5° to 5°, step size of 0.01° with a time of 0.3 s, the slit used was 0.1 mm for divergent and 0.3 mm for convergent, 0.5 for central. N_2_ adsorption and desorption were used to provide the textural properties, and the surface area was obtained using the B.E.T. method, mesoporous volume (V_meso_), microporous volume (V_micro_), and external surface area (Sext) by the t-Plot method [56]. The details are presented in the Supplementary Materials. Fourier transform infrared spectroscopy (FTIR) and nuclear magnetic resonance (NMR) for Si^29^ were used to evaluate the groups present in the material and the structure of the material, respectively. Scanning electron microscopy (SEM) obtained morphology images of SBA-15 material. Energy dispersive spectroscopy (EDS) was performed to obtain data on the chemical composition of the material. All details are shown in the Supplementary Materials.

### 3.4. Affinity Test

The tests were performed using a 1.5 g L^−1^ to dosage of SBA-15 and 10 mL of the BPA, CIP, and LS solutions (initial concentration = 0.2 mmol L^−1^) at pH 5, 25 °C and 200 rpm. The mixture was kept under constant stirring for 48 h, at 25 °C and 200 rpm, and the tests were performed in triplicate. The aliquots were filtered and quantified using the UV-Vis spectrophotometer. The removal percentage (%R, Equation (1)) and the adsorption capacity (q, mmol g^−1^, Equation (2)) were obtained using their equations. Details about the affinity tests can be found in the Supplementary Materials.

### 3.5. Determination of pH_ZPC_ and Zeta Potential

The pH_ZPC_ was determined using NaOH solution as an electrolyte and HCl for pH values in the range of 1 to 11. This solution was separated into different Erlenmeyer with SBA-15, a dosage of 1.5 g L^−1^, and stirring of 200 rpm for 24 h at 25 °C. The pH was measured initially (pH_i_) and at the end (pH_f_) after the adsorption process. Afterward, the zeta potential was measured at each final pH for all samples, described in Supplementary Materials.

### 3.6. Kinetics, Equilibrium Isotherms, and Thermodynamic Studies

The tests were performed with the best system of SBA-15 + emerging contaminant, a dosage of 1.5 g L^−1^ and quantification was performed in the same way as for the affinity tests. Adsorption kinetics were evaluated at 0.15, 0.30 and 0.50 mmol g^−1^. The kinetics models comprise pseudo-first order (PFO) (Equation (1)) [57], pseudo-second order (PSO) (Equation (2)) [58], interparticle diffusion (IPD) (Equation (3)) [59], and external mass transfer resistance (EMTR) (Equations (4) and (5)) [60]. Equilibrium isotherms were performed in the C_0_ range from 0.01 to 1 mmol g^−1^ at 15 °C, 25 °C, and 35 °C. Details of the following equations, the Langmuir (Equation (6)) [61], Freundlich (Equation (7)) [62], and B.E.T. (Equation (8)) [63], are shown in the Supplementary Materials.

(1) $$q_{t}=q_{e}(1-e^{K_{1}t})$$

(2) $$q_{t}=\frac{K_{2}q_{e}^{2}t}{1+K_{2}q_{e}t}$$

(3) $$q_{t}=K_{I}t^{\frac{1}{2}}+C$$

(4) $$\frac{{dC}_{f}}{dt}=\frac{K_{TM}V}{{mq}_{m}K_{L}}{\left(1+K_{L}C_{F}\right)}^{2}(C-C_{f})$$

(5) $$\frac{dC}{dt}=-K_{TM}(C-C_{f})$$

(6) $$q_{e}=\frac{q_{Max}K_{L}C_{e}}{1+K_{L}C_{e}}$$

(7) $$q_{e}=K_{F}C_{E}^{1/n}$$

(8) $$q_{e}=\frac{q_{B}K_{B}C_{e}}{\left(1-K_{U}C_{e})(1-K_{U}C_{e}+K_{B}C_{e}\right)}$$

The thermodynamic analysis was conducted to comprehend the behavior of the adsorption process under varying temperature conditions. The equilibrium thermodynamic constant (K_D_) was calculated, and the necessary modifications were made for use in calculating the parameters [64]. All values of the thermodynamic parameters of entropy variation (ΔS°), enthalpy variation (ΔH°), activation energy (E_a_), and Gibbs energy variation (ΔG°) were calculated. Details are presented in the Supplementary Materials.

### 3.7. The Simplified Batch Design

The mass quantity of SBA-15 for adsorption of the emerging contaminant at different removal percentages was made available using the simplified batch design. First, the mass balance for the system was calculated using Equations (S15) and (S16) in Supplementary Materials [45]. Considering the best model fitted with the experimental equilibrium data is important. The mass required to remove 50–90% of the contaminant with a value of 0.2 mmol L^−1^ to initial concentration and a solution volume ranging from 1 to 10 L was calculated.

## 4. Conclusions

The SBA-15 material was successfully synthesized, exhibiting an ordered structure and excellent textural properties. The characteristic peaks of symmetry p6mm were observed in the XRD spectrum. Furthermore, the FTIR and NMR spectra confirmed the presence of silica structure condensed and silanol group in the structure of the synthesized material. The SBET and DP values were 688.7 m^2^ g^−1^ and 6.0 nm, respectively. The synthesized SBA-15 presented a fiber morphology, which is common for this material. The use of the synthesized SBA-15 as an adsorbent material was evaluated. Affinity tests were performed with contaminant bisphenol A, ciprofloxacin, and losartan. Better results were found for the SBA-15 + CIP system, which can be attributed to the better interaction of CIP on Si-O-Si and Si-OH sites present on the surface. For this reason, this system was chosen to continue the adsorption tests. The kinetic tests showed an equilibrium time of 20 min and the PSO kinetic model had the best fit. The equilibrium tests demonstrated that temperature can negatively influence the process since the *q_max_* decreases with the increasing temperature. This behavior may also indicate an exothermic system. The B.E.T. model showed the best fit, suggesting multilayer adsorption with varying interaction sites. The simplified batch design demonstrated that 27 g of SBA-15 is necessary to achieve 90% CIP removal in a solution volume of 10 L. Finally, it is possible to conclude that the synthesized material presented satisfactory performance in the adsorption process of ciprofloxacin from aqueous solutions.

## Figures and Tables

**Table 1:** Textural properties for mesoporous silica material.

Material	S_BET_ (m^2^/g)	V_meso_ (cm^3^/g)	V_micro_ (cm^3^/g)	S_ext_ (m^2^/g)	D_BJH_ (nm)
SBA-15	688.7	0.731	0.073	504.8	6.0

S_BET_ = Surface area by BET method; Vmeso = mesopore volume; Vmicro = micropore volume; Sext = external surface area by t-Plot method. D_BJH_: pore diameter by BJH method.

**Table 2:** Parameters obtained from ^29^Si for SB-FIB.

Material	Signal	δ (ppm)	Area (%)
SBA-15	Q^2^	−91.84	5.73
	Q^3^	−101.54	30.48
	Q^4^	−109.09	63.79

**Table 3:** Parameters for the PFO, PSO, IPD, and EMTR kinetic models.

Models	Parameters	(C_0_) (mmol L^−1^)		
		0.10	0.30	0.50
	q_e_	0.066	0.109	0.132
PFO	q*_PFO_*	0.060	0.102	0.122
	k_1_	2.196	3.568	5.660
	R^2^_Adj_	0.952	0.957	0.982
	AICc	−124.559	−113.199	−105.539
	MRD (%)	3.54	0.60	0.83
PSO	q*_PSO_*	0.061	0.106	0.126
	k_2_	8.346	7.872	23.403
	R^2^_Adj_	0.955	0.982	0.986
	AICc	−87.749	−76.599	−79.779
	MRD (%)	3.46	1.35	1.06
IPD	K_i_	0.00264	0.00517	0.00389
	c	0.0537	0.0862	0.115
	R^2^_Adj_	0.8678	0.859	0.764
	AICc	−83.843	−60.403	−49.434
	MRD (%)	4.31	8.01	18.99
EMTR	K_TM_	1.441	1.774	2.188
	R^2^	0.978	0.980	0.992
	AICc	−128.222	−116.880	−123.290
	MRD (%)	5.4	5.1	3.1

where equilibrium adsorption capacity (*q_e_*): mmol g^−1^, equilibrium adsorption capacity for the PFO model (*q_PFO_*): mmol g^−1^, the pseudo-first order constant (k_1_): min^−1^, equilibrium adsorption capacity to PFO model (*q_PSO_*): mmol g^−1^, pseudo-second order constant (k_2_): L mol^−1^ min^−1^, the constant for the intraparticle diffusion model (Ki): mmol L^−1^ min^−1^; constant related to the thickness of the boundary layers (c): mmol L^−1^, external mass transfer resistance (K_TM_): min^−1^.

**Table 4:** Isothermal models and parameters obtained.

Models	Parameters	Temperature (°C)		
		15	25	35
	*q_max_*	0.374	0.207	0.208
Langmuir	*q_L_*	0.450	0.207	0.220
	K_L_	1.477	2.249	2.705
	R^2^_Adj_	0.674	0.697	0.809
	AICc	−44.858	−55.204	−58.524
	MRD (%)	35.32	38.17	27.41
Freundlich	K_F_	0.693	0.189	0.244
	1/n	1.544	0.876	0.971
	R^2^_Adj_	0.788	0.645	0.919
	AICc	−36.547	−35.644	−50.798
	MRD (%)	28.44	29.44	25.51
BET	*q_b_*	0.059	0.041	0.068
	K_b_	121.581	449.752	25.871
	K_u_	1.434	0.954	0.818
	R^2^_Adj_	0.933	0.905	0.944
	AICc	−62.457	−63.816	−71.705
	MRD (%)	19.66	19.56	11.53

where adsorption capacity from experimental data (*q_max_*): mmol g^−1^, maximum monolayer adsorption capacity to Langmuir model (*q_L_*): mmol g^−1^, Langmuir model constant (K_L_): L mmol^−1^, Freundlich model constant (K_F_): (mmol g^−1^) (L mol^−1^)^1/n^, maximum monolayer adsorption capacity for B.E.T. model (*q_b_*): mmol g^−1^, monolayer adsorption constant (Kb): L g^−1^, multilayer adsorption constant (K_u_): L g^−1^.

**Table 6:** Thermodynamic parameters for experimental data.

T (°C)	ΔH° (kJ mol^−1^)	ΔS° (J mol^−1^ K^−1^)	ΔG° (kJ mol^−1^)	E_a_ (kJ mol^−1^)
15	−55.380	−89.208	−29.675	−52.902
25			−28.783	−52.819
35			−27.819	−52.298

Entropy variation (ΔS°); enthalpy variation (ΔH°); activation energy (E_a_); Gibbs energy variation (ΔG°).

## Data Availability

Data are contained within the article.

## Supplementary Materials

The following supporting information can be downloaded at: https://www.mdpi.com/article/10.3390/molecules30051040/s1, S1—adsorbent characterization; S2—affinity test; S3—Zeta potential; S4—kinetics, equilibrium models, and thermodynamic study; S5—the simplified batch design; Figure S1—color map obtained by EDS for C (carbon ribbon), Au (coating), Si, and O (SBA-15); Figure S2—EDS spectrum for SBA-15 material; Figure S3—thermodynamic plot of lnK_c_ versus 1/T.

## References

[1] Solliec M., Roy-Lachapelle A., Gasser M.-O., Coté C., Généreux M., Sauvé S. (2016). Fractionation and Analysis of Veterinary Antibiotics and Their Related Degradation Products in Agricultural Soils and Drainage Waters Following Swine Manure Amendment. Sci. Total Environ..

[2] Taheran M., Naghdi M., Brar S.K., Verma M., Surampalli R.Y. (2018). Emerging Contaminants: Here Today, There Tomorrow!. Environ. Nanotechnol. Monit. Manag..

[3] Veetil D.P., Mercier G., Blais J.F., Chartier M., Tran L.H., Taillard V. (2014). Remediation of Contaminated Dredged Sediments Using Physical Separation Techniques. Soil Sediment Contam..

[4] Ahmed S.F., Mofijur M., Nuzhat S., Chowdhury A.T., Rafa N., Uddin M.A., Inayat A., Mahlia T.M.I., Ong H.C., Chia W.Y. (2021). Recent Developments in Physical, Biological, Chemical, and Hybrid Treatment Techniques for Removing Emerging Contaminants from Wastewater. J. Hazard. Mater..

[5] Kumar R., Qureshi M., Vishwakarma D.K., Al-Ansari N., Kuriqi A., Elbeltagi A., Saraswat A. (2022). A Review on Emerging Water Contaminants and the Application of Sustainable Removal Technologies. Case Stud. Chem. Environ. Eng..

[6] Baek M.K., Sung T.I., Cho E.S., Namkung K.C., Bae D.J., Park I.H. (2012). Magnetic Separation for Contaminants in Wastewater Using Magnetic Micro Bead. IEEE Trans. Magn..

[7] Abdi J., Banisharif F., Khataee A. (2021). Amine-Functionalized Zr-MOF/CNTs Nanocomposite as an Efficient and Reusable Photocatalyst for Removing Organic Contaminants. J. Mol. Liq..

[8] Bolong N., Ismail A.F., Salim M.R., Matsuura T. (2009). A Review of the Effects of Emerging Contaminants in Wastewater and Options for Their Removal. Desalination.

[9] Esfandiar N., Suri R., McKenzie E.R. (2022). Competitive Sorption of Cd, Cr, Cu, Ni, Pb and Zn from Stormwater Runoff by Five Low-Cost Sorbents; Effects of Co-Contaminants, Humic Acid, Salinity and PH. J. Hazard. Mater..

[10] Akinnawo S.O. (2023). Eutrophication: Causes, Consequences, Physical, Chemical and Biological Techniques for Mitigation Strategies. Environ. Chall..

[11] Kebede G., Tafese T., Abda E.M., Kamaraj M., Assefa F. (2021). Factors Influencing the Bacterial Bioremediation of Hydrocarbon Contaminants in the Soil: Mechanisms and Impacts. J. Chem..

[12] Pérez-Botella E., Valencia S., Rey F. (2022). Zeolites in Adsorption Processes: State of the Art and Future Prospects. Chem. Rev..

[13] Alomar T., Qiblawey H., Almomani F., Al-Raoush R.I., Han D.S., Ahmad N.M. (2023). Recent Advances on Humic Acid Removal from Wastewater Using Adsorption Process. J. Water Process Eng..

[14] Wang Y., Zhang F., Wang Y., Ren J., Li C., Liu X., Guo Y., Guo Y., Lu G. (2009). Synthesis of Length Controllable Mesoporous SBA-15 Rods. Mater. Chem. Phys..

[15] Silva F.E.d., Rigoti E., Mello M.I.S.d., Pergher S.B.C. (2024). Tuning Textural Properties by Changing the Morphology of SBA-15 Mesoporous Materials. Materials.

[16] Zholobenko V.L., Khodakov A.Y., Impéror-Clerc M., Durand D., Grillo I. (2008). Initial Stages of SBA-15 Synthesis: An Overview. Adv. Colloid. Interface Sci..

[17] Laskowski Ł., Laskowska M., Walcarius A., Doskocz M., Vila N., Karczmarska A., Pawlik P., Goraus J., Balin K., Dulski M. (2024). Synthesis and Characterization of SBA-15 Silica Containing Cyclam inside Pores for Capturing Iron Chloride: Analysis of Interactions between Ferrous Chloride and Cyclam in a System with Strongly Dispersed Functional Groups on the SiO_2_ Surface. Microporous Mesoporous Mater..

[18] Thommes M., Kaneko K., Neimark A.V., Olivier J.P., Rodriguez-Reinoso F., Rouquerol J., Sing K.S.W. (2015). Physisorption of Gases, with Special Reference to the Evaluation of Surface Area and Pore Size Distribution (IUPAC Technical Report). Pure Appl. Chem..

[19] Ojeda-López R., Pérez-Hermosillo I.J., Marcos Esparza-Schulz J., Cervantes-Uribe A., Domínguez-Ortiz A. (2015). SBA-15 Materials: Calcination Temperature Influence on Textural Properties and Total Silanol Ratio. Adsorption.

[20] Genç N., Dogan E.C. (2015). Adsorption Kinetics of the Antibiotic Ciprofloxacin on Bentonite, Activated Carbon, Zeolite, and Pumice. Desalin. Water Treat..

[21] Avcı A., İnci İ., Baylan N. (2019). A Comparative Adsorption Study with Various Adsorbents for the Removal of Ciprofloxacin Hydrochloride from Water. Water Air Soil Pollut..

[22] Bizi M., El Bachra F.E. (2020). Evaluation of the Ciprofloxacin Adsorption Capacity of Common Industrial Minerals and Application to Tap Water Treatment. Powder Technol..

[23] Macías-Ferrer D., Melo-Banda J.A., Silva-Rodrigo R., Páramo-García U., Verde-Gómez J.Y., Del-Angel-Vicente P. (2018). Synthesis of Micro/Nanostructured Carbon from Refined Sugar and Its Electrochemical Performance. Int. J. Electrochem. Sci..

[24] Rekos K., Kampouraki Z.C., Sarafidis C., Samanidou V., Deliyanni E. (2019). Graphene Oxide Based Magnetic Nanocomposites with Polymers as Effective Bisphenol-A Nanoadsorbents. Materials.

[25] de Andrade J.R., Oliveira M.F., Canevesi R.L.S., Landers R., da Silva M.G.C., Vieira M.G.A. (2020). Comparative Adsorption of Diclofenac Sodium and Losartan Potassium in Organophilic Clay-Packed Fixed-Bed: X-Ray Photoelectron Spectroscopy Characterization, Experimental Tests and Theoretical Study on DFT-Based Chemical Descriptors. J. Mol. Liq..

[26] Roca Jalil M.E., Baschini M., Sapag K. (2015). Influence of PH and Antibiotic Solubility on the Removal of Ciprofloxacin from Aqueous Media Using Montmorillonite. Appl. Clay Sci..

[27] Li H., Zhang D., Han X., Xing B. (2014). Adsorption of Antibiotic Ciprofloxacin on Carbon Nanotubes: PH Dependence and Thermodynamics. Chemosphere.

[28] Xing X., Feng J., Lv G., Song K., Mei L., Liao L., Wang X., Xu B. (2015). Adsorption Mechanism of Ciprofloxacin from Water by Synthesized Birnessite. Adv. Mater. Sci. Eng..

[29] Bui T.X., Choi H. (2009). Adsorptive Removal of Selected Pharmaceuticals by Mesoporous Silica SBA-15. J. Hazard. Mater..

[30] Kokunešoski M., Gulicovski J., Matović B., Logar M., Milonjić S.K., Babić B. (2010). Synthesis and Surface Characterization of Ordered Mesoporous Silica SBA-15. Mater. Chem. Phys..

[31] da Silva Marques V.B., dos Santos A.G., de Lima Leite R.H., dos Santos F.K.G. (2020). Functionalization of SBA-15 with EDTA and Its Application in Removing Ca2+ and Mg2+ Ions from Hard Water. Rev. Verde Agroecol. E Desenvolv. Sustentável.

[32] Ezzeddine Z., Batonneau-Gener I., Pouilloux Y., Hamad H., Saad Z. (2021). The Applicability of as Synthesized Mesoporous Carbon CMK-3 as a Heavy Metals Adsorbent: Application to Real Water Samples. Energy Sources Part A Recovery Util. Environ. Eff..

[33] da Silva de Assis D.A., dos Santos E.G., Eiras D. (2024). Efficient and Reusable Mesoporous Silica Structures for Ciprofloxacin Removal from Water Media. J. Water Process Eng..

[34] Peng X., Hu F., Huang J., Wang Y., Dai H., Liu Z. (2016). Preparation of a Graphitic Ordered Mesoporous Carbon and Its Application in Sorption of Ciprofloxacin: Kinetics, Isotherm, Adsorption Mechanisms Studies. Microporous Mesoporous Mater..

[35] Khan A.H., Abdul Aziz H., Palaniandy P., Naushad M., Zouli N. (2024). Ciprofloxacin Adsorption onto CNT Loaded Pumice: Adsorption Modelling, Kinetics, Equilibriums and Reusability Studies. J. Mol. Liq..

[36] Mao H., Wang S., Lin J.Y., Wang Z., Ren J. (2016). Modification of a Magnetic Carbon Composite for Ciprofloxacin Adsorption. J. Environ. Sci..

[37] Wu Y., Zheng H., Li H., Sun Y., Zhao C., Zhao R., Zhang C. (2021). Magnetic Nickel Cobalt Sulfide/Sodium Dodecyl Benzene Sulfonate with Excellent Ciprofloxacin Adsorption Capacity and Wide PH Adaptability. Chem. Eng. J..

[38] Tran Q.T., Do T.H., Ha X.L., Nguyen H.P., Nguyen A.T., Ngo T.C.Q., Chau H.D. (2022). Study of the Ciprofloxacin Adsorption of Activated Carbon Prepared from Mangosteen Peel. Appl. Sci..

[39] Wu M., Zhao S., Jing R., Shao Y., Liu X., Lv F., Hu X., Zhang Q., Meng Z., Liu A. (2019). Competitive Adsorption of Antibiotic Tetracycline and Ciprofloxacin on Montmorillonite. Appl. Clay Sci..

[40] Antonelli R., Malpass G.R.P., da Silva M.G.C., Vieira M.G.A. (2020). Adsorption of Ciprofloxacin onto Thermally Modified Bentonite Clay: Experimental Design, Characterization, and Adsorbent Regeneration. J. Environ. Chem. Eng..

[41] Lu D., Xu S., Qiu W., Sun Y., Liu X., Yang J., Ma J. (2020). Adsorption and Desorption Behaviors of Antibiotic Ciprofloxacin on Functionalized Spherical MCM-41 for Water Treatment. J. Clean. Prod..

[42] Módenes A.N., Bazarin G., Borba C.E., Locatelli P.P.P., Borsato F.P., Pagno V., Pedrini R., Trigueros D.E.G., Espinoza-Quiñones F.R., Scheufele F.B. (2021). Tetracycline Adsorption by Tilapia Fish Bone-Based Biochar: Mass Transfer Assessment and Fixed-Bed Data Prediction by Hybrid Statistical-Phenomenological Modeling. J. Clean. Prod..

[43] de Oliveira Carvalho C., Costa Rodrigues D.L., Lima É.C., Santanna Umpierres C., Caicedo Chaguezac D.F., Machado Machado F. (2019). Kinetic, Equilibrium, and Thermodynamic Studies on the Adsorption of Ciprofloxacin by Activated Carbon Produced from Jerivá (*Syagrus romanzoffiana*). Environ. Sci. Pollut. Res..

[44] Song X., Zhang Y., Yan C., Jiang W., Chang C. (2013). The Langmuir Monolayer Adsorption Model of Organic Matter into Effective Pores in Activated Carbon. J. Colloid. Interface Sci..

[45] de Almeida A.S.V., Mastelaro V.R., da Silva M.G.C., Prediger P., Vieira M.G.A. (2022). Adsorption of 17α-Ethinylestradiol onto a Novel Nanocomposite Based on Graphene Oxide, Magnetic Chitosan and Organoclay (GO/MCS/OC): Kinetics, Equilibrium, Thermodynamics and Selectivity Studies. J. Water Process Eng..

[46] Sert Çok S., Koç F., Len A., Almásy L., Dudás Z. (2025). Silica Aerogels Modified with Vinyl, Epoxide, Methacrylate Moieties for the Removal of Ciprofloxacin by Adsorption from Water. Sep. Purif. Technol..

[47] Hamadeen H.M., Elkhatib E.A. (2022). New Nanostructured Activated Biochar for Effective Removal of Antibiotic Ciprofloxacin from Wastewater: Adsorption Dynamics and Mechanisms. Environ. Res..

[48] Afzal M.Z., Sun X.F., Liu J., Song C., Wang S.G., Javed A. (2018). Enhancement of Ciprofloxacin Sorption on Chitosan/Biochar Hydrogel Beads. Sci. Total Environ..

[49] Ma W., Dai J., Dai X., Yan Y. (2014). Preparation and Characterization of Chitosan/Kaolin/Fe_3_O_4_ Magnetic Microspheres and Their Application for the Removal of Ciprofloxacin. Adsorpt. Sci. Technol..

[50] Huš M., Dasireddy V.D.B.C., Strah Štefančič N., Likozar B. (2017). Mechanism, Kinetics and Thermodynamics of Carbon Dioxide Hydrogenation to Methanol on Cu/ZnAl2O4 Spinel-Type Heterogeneous Catalysts. Appl. Catal. B.

[51] Azhagiya Singam E.R., Zhang Y., Magnin G., Miranda-Carvajal I., Coates L., Thakkar R., Poblete H., Comer J. (2019). Thermodynamics of Adsorption on Graphenic Surfaces from Aqueous Solution. J. Chem. Theory Comput..

[52] Yin D., Xu Z., Shi J., Shen L., He Z. (2018). Adsorption Characteristics of Ciprofloxacin on the Schorl: Kinetics, Thermodynamics, Effect of Metal Ion and Mechanisms. J. Water Reuse Desalin..

[53] Uzosike A.O., Ofudje E.A., Adeogun A.I., Akinyele J.O., Idowu M.A. (2022). Comparative Analysis of Bisphenol-A Removal Efficiency from Water: Equilibrium, Kinetics, Thermodynamics and Optimization Evaluations. J. Iran. Chem. Soc..

[54] de Sá Costa H.P., Duarte E.D.V., da Silva M.G.C., Vieira M.G.A. (2024). Adsorption of Diclofenac and Losartan Using Multi-Walled Carbon Nanotubes Functionalized with Iron Nanoparticles via the Green Route: Equilibrium, Thermodynamics, and Machine Learning Studies. J. Water Process Eng..

[55] Zhao D., Feng J., Huo Q., Melosh N., Fredrickson G.H., Chmelka B.F., Stucky G.D. (1998). Triblock Copolymer Syntheses of Mesoporous Silica with Periodic 50 to 300 Angstrom Pores. Science.

[56] Galarneau A., Cambon H., Di Renzo F., Ryoo R., Choi M., Fajula F. (2003). Microporosity and Connections between Pores in SBA-15 Mesostructured Silicas as a Function of the Temperature of Synthesis. New J. Chem..

[57] Lagergren S. (1907). Zur Theorie Der Sogenannten Adsorption Gelöster Stoffe. Zeitschr Für Chem. Und Ind. Der Kolloide.

[58] Ho Y.S., McKay G. (1998). A Comparison of Chemisorption Kinetic Models Applied to Pollutant Removal on Various Sorbents. Process Saf. Environ. Prot..

[59] Weber W.J., Morris J.C. (1963). Kinetics of Adsorption on Carbon from Solution. J. Sanit. Eng. Div..

[60] Puranik P.R., Modak J.M., Paknikar K.M. (1999). A Comparative Study of the Mass Transfer Kinetics of Metal Biosorption by Microbial Biomass.

[61] Langmuir I. (1918). The Adsorption of Gases on Plane Surfaces of Glass, Mica and Platinum. J. Am. Chem. Soc..

[62] Haring M.M. (1926). Colloid and Capillary Chemistry (Freundlich, Herbert). J. Chem. Educ..

[63] Ebadi A., Soltan Mohammadzadeh J.S., Khudiev A. (2009). What Is the Correct Form of BET Isotherm for Modeling Liquid Phase Adsorption?. Adsorption.

[64] Milonjić S.K. (2007). A Consideration of the Correct Calculation of Thermodynamic Parameters of Adsorption. J. Serbian Chem. Soc..

