# Supplementary material for: Ordered Mesopore Channels of SBA-15 for Contaminant Adsorption: Characterization, Kinetic, Equilibrium, and Thermodynamic Studies
Source: Molecules. 2025 Feb 24;30(5):1040. doi: 10.3390/molecules30051040 (PMC11902132; doi:10.3390/molecules30051040)
Supplement: Supplementary file 1 [file molecules-30-01040-s001.zip › molecules-3475879-supplementary.pdf]

SUPPLEMENTARY MATERIAL

Ordered Mesoporous Channels of SBA-15 for Contaminant Adsorption: Characterization, Kinetic, Equilibrium and Thermodynamic Studies

Francisco Emanuel da Silva[a], Marcela Pires Spaolonzi [b], Melissa Gurgel Adeodato Vieira [b], Sibele Berenice Castellã Pergher[a,*]

[a] Institute of Chemistry, Universidade Federal do Rio Grande do Norte, Senador Salgado Filho Av., 3000, Natal, Rio Grande do Norte, Brazil.

[b] School of Chemical Engineering, Universidade Estadual de Campinas, Albert Einstein Av., 500, Campinas, São Paulo, Brazil

*Correspondent author: sibelepergher@gmail.com

S1 – Adsorbent characterization

To evaluate the ordered structure of SBA-15, X-ray diffraction was used on a Bruker D2Phaser equipped with a Lynxeyer detector (Stockholm, Sweden), copper radiation (CuKα λ = 1.54 Å), Ni filter, 10 mA current, and 30 kV voltage. Textural properties were evaluated with N_2 adsorption and desorption using a Micromeritics ASAP 2020 instrument (Norcross, GA, USA). The method was based on the N_2 condensation temperature of 77 K. Pretreatment was performed at 150 °C for 2 h under vacuum. Fourier transform infrared spectroscopy (FTIR) was obtained with a Shimadzu IRAffinity1 Fourier transform infrared spectrophotometer (Kyoto, Japan) in the range of 4000 to 400 cm-1.

Nuclear magnetic resonance imaging was performed using a Bruker Avance III/HD 400MHz spectrometer (Stockholm, Sweden) with 79.41 MHz solid-state, 10kHz rotation frequency, and 9.4 T magnetic field, kaolin as external reference (chemical shift 91.26 ppm), 64 1H spinal decoupling pulse, 60 s pulse delay. Morphology images were obtained using a TESCAN MIRA 4 scanning electron microscope (Brno, Czech Republic), a beam secondary electron detector, 10 KeV energy. EDS analyzes were carried out using aztec software coupled to an Ultim Max 20 Kev EDS detector, Oxford Instruments. To guide the points of the EDS analysis, backscattered electron images

(BSE) were used. The sample was coated with a gold layer using a Denton Vacuum Desk evaporator model V (Moorestown, NJ, USA), voltage 30 for 60 s.

S2 – Affinity test

For quantitative analyses, a Shimadzu/UVmini-1240 UV-Vis spectrophotometer was used in triplicate samples, with wavelengths of 276 nm (CIP and BPA) and 205 nm (LS). The removal percentage and adsorption capacity were calculated using Eq. S1 and Eq. S2, respectively:

$$\%R = (C_0 - C_t / C_0)\ 100 \quad \text{(S1)}$$

$$q = (V / m)\ (C_0 - C_t) \quad \text{(S2)}$$

C_t: concentration of the solution at time t (mmol L^{-1}); C_0: initial concentration of the solution (mmol L^{-1}); m: mass of the adsorbent (g); V: volume of solution (L).

S3 – Zeta potential

The final pH zeta potential was measured using the Stabino® II particle charge titration model from Colloid Metrix. A 0.4 groove piston (400 μm gap) was used.

S4 – Kinetics, equilibrium models and Thermodynamic study

The pseudo-first order, pseudo-second order, linear interparticle diffusion and external mass transfer resistance (EMTR) model were applied to experimental data using Eqs. 3, 4, 5 and 6–7, respectively.

$$q_t = q_e(1 - e^{K_1 t}) \quad \text{(S3)}$$

$$q_t = \frac{K_2 q_e^2 t}{1 + K_2 q_e t} \quad \text{(S4)}$$

$$q_t = K_I t^{\frac{1}{2}} + Ci \quad \text{(S5)}$$

$$\frac{dC_f}{dt} = \frac{K_{TM} V}{m q_m K_L}(1 + K_L C_F)^2 (C - C_f) \quad \text{(S6)}$$

$$\frac{dC}{dt} = -K_{TM}(C - C_f) \quad \text{(S7)}$$

q_e is the equilibrium adsorption capacity (mmol g^{-1}), t is time (min), C_f is the concentration after adsorption process (mmol L^{-1}), Ci is the initial concentration (mmol L^{-1}), , K_1 is the pseudo-first order constant (min^{-1}), K_2 is the pseudo-second order constant (g $mmol^{-1}$ min^{-1}), K_I is the constant for intraparticle diffusion model (mmol g^{-1} min^{-1}), K_{TM} is the external film mass transfer coefficient (min^{-1}) and C is constant related to limit layers thickness (mmol g^{-1}).

Equilibrium models applied: Langmuir, Eq. (S8), Freundlich, Eq. (S9) and B.E.T., Eq. (S10), the models were adjusted to the data obtained.

$$q_e = \frac{q_{Max} K_L C_e}{1+K_L C_e} \quad \text{(S8)}$$

$$q_e = K_F C_E^{1/n} \quad \text{(S9)}$$

$$q_e = \frac{q_B K_B C_e}{(1-K_U C_e)(1-K_U C_e+K_B C_e)} \quad \text{(S10)}$$

For the Freundlich model: K_F is the model constant [(mmol g^{-1})(L mol^{-1}) $^{1/n\ n}$], n is assigned the exponent. For the Langmuir model: q_L is the maximum monolayer adsorption capacity (mmol g^{-1}), K_L is the model constant (L $mmol^{-1}$). For the B.E.T. model: q_B is the maximum monolayer adsorption capacity (mmol g^{-1}), K_B is the monolayer adsorption constant (L g^{-1}), K_U is the multilayer adsorption constant (L g^{-1}).

The thermodynamic study was performed using the equilibrium thermodynamic constant (K_D) determined by Henry's law, applied in the infinite dilution region of each temperature (Eq. S11). The modification proposed by Milonjic was used seeking to make dimensionless for application in the Van't Hoff equation (Eq. S13). The value for the parameters of Gibbs energy variation (ΔG^0), enthalpy variation (ΔH^0), entropy variation (ΔS^0) and activation energy (E_a) were determined using the Eqs. (S12), (S13) and (S14):

$$q_e = K_D C_e \quad \text{(S11)}$$

$$\Delta G^0 = -RTln(K_C) \quad \text{(S12)}$$

$$ln\, ln\, (K_C) = \frac{\Delta S^0}{R} - \frac{\Delta H^0}{RT}$$

(13)

$$E_a = \Delta H^0 + RT \quad \text{(S14)}$$

K_C: dimensionless thermodynamic equilibrium constant, R: the ideal gas constant, and T (K): the experimental temperatures.

S5 - The simplified batch design

The calculation was performed based on a mass balance for this system and is described in Eq. S15 [25]:

$$V(C_0 - C_1) = m(q_1 - q_0) \tag{S15}$$

Some considerations are necessary, the absence of emerging contaminants in SBA-15, accounting for the concentration in the fluid phase being the same as that of equilibrium, and considering the amount absorbed in the solid phase as what was retained at equilibrium. Eq. S15 transforms Eq. S16.

$$V(C_0 - C_e) = mq_e \tag{S16}$$

The mass of mesoporous silica required for solution volumes between 1 and 10 L, at an initial concentration of 0.2 mmol L^{-1}, was calculated and evaluated for removal efficiencies of 50%, 60%, 70%, 80%, and 90%. The data, obtained from the equilibrium isotherm at 25°C, were used to determine the model that best fitted the experimental results for this system.

S6 – Characterization of the nanostructured mesoporous material SBA-15

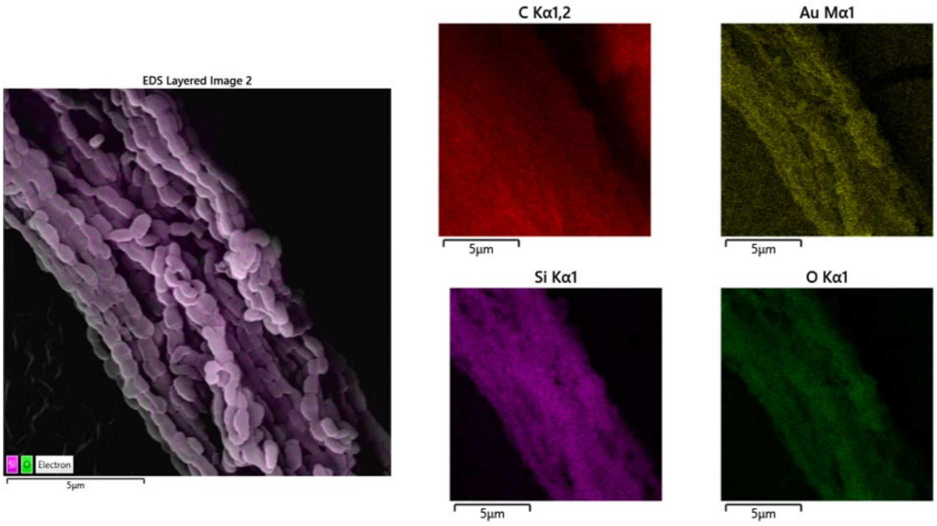

Figure S1 - Color map obtained by EDS for C (carbon ribbon), Au (coating), Si and O (SBA-15).

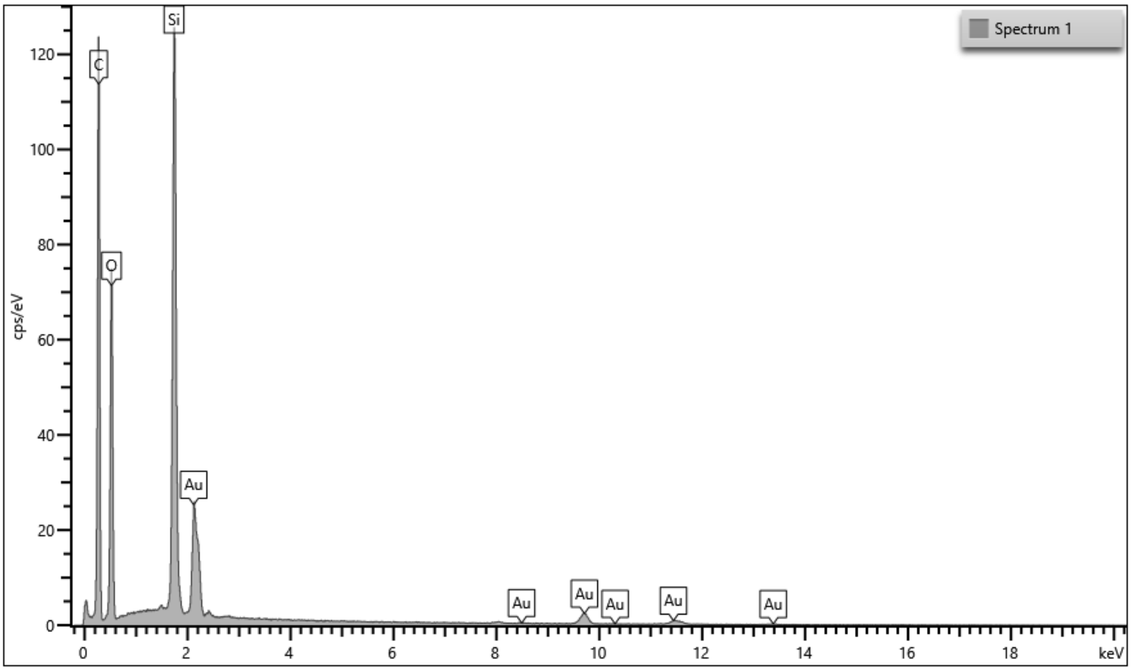

Figure S2 – EDS spectrum for SBA-15 material.

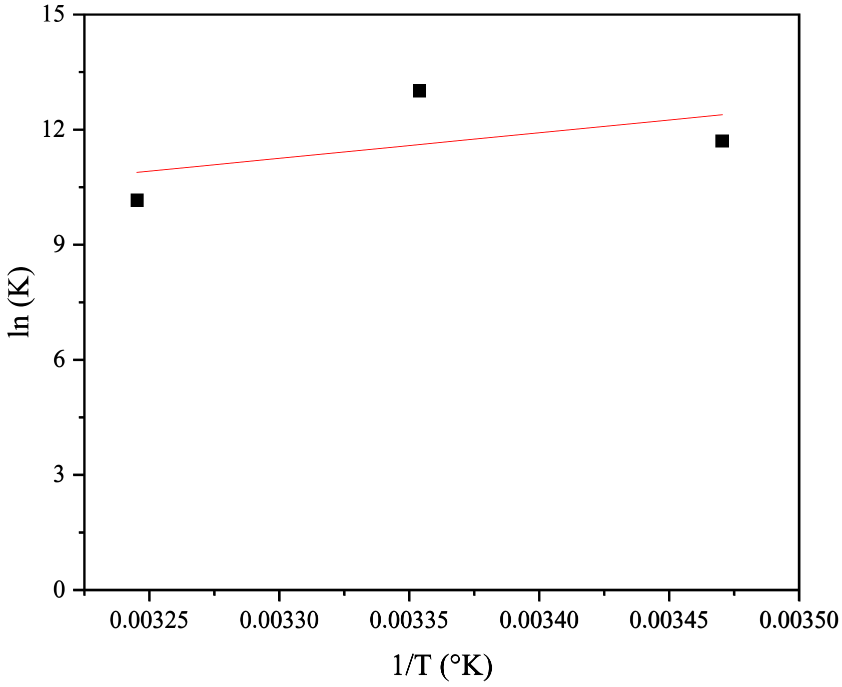

Figure S3 – Thermodynamic plot of lnK_c versus 1/T.
